# Nutritional supplementation with *Panax ginseng* extract and bone health in osteoporotic animal models: a systematic review and meta-analysis

**DOI:** 10.3389/fnut.2026.1748861

**Published:** 2026-06-03

**Authors:** Rui Tang, Dongping Wan, Haoxiang Yuan, Chuan Leng, Feilong Li, Rui Wang, Xiang Ji, Shihang Cao, Xi Gao

**Affiliations:** 1 Honghui Hospital, Xi'an Jiaotong University, Xi‘an, Shanxi, China; 2 The Clinical Medical College, Chengdu University of Chinese Traditional Medicine, Chengdu, Sichuan, China; 3 The First Clinical Medical College, Guangxi University of Chinese Medicine, Nanning, China; 4 Department of Orthopedics and Traumatology, The Affiliated Traditional Chinese Medicine Hospital, Southwest Medical University, Luzhou, Sichuan, China

**Keywords:** bone mineral density, meta-analysis, osteoporosis, *Panax ginseng*, traditional Chinese medicine

## Abstract

**Purpose:**

To systematically evaluate the anti-osteoporotic efficacy of *Panax ginseng* extract as a nutritional supplement in animal models and provide high-quality evidence.

**Methods:**

Eight databases were searched for randomized controlled trials comparing *Panax ginseng* extract with placebo in osteoporotic animals. Data on bone mineral density (BMD), bone microarchitecture, biomechanical parameters, and biochemical markers were extracted. Risk of bias (RoB) was assessed using SYRCLE's tool. Standardized mean differences (SMDs) or mean differences (MDs) with 95% confidence intervals (CIs) were calculated. Heterogeneity was evaluated with *I*^2^ statistics, and sensitivity and subgroup analyses were performed.

**Results:**

Twenty-eight studies met inclusion criteria. *Panax ginseng* extract significantly increased BMD (*SMD* = 2.21, 95% *CI* 1.62–2.79, *p* < 0.000001) and improved trabecular number, thickness, bone volume fraction (BV/TV), and reduced trabecular separation. Biomechanical properties, including maximum load and stiffness, were enhanced. Biochemical analyses showed increased procollagen type I N-terminal propeptide (PINP), estradiol, osteocalcin, serum calcium, and phosphate, with decreased tartrate-resistant acid phosphatase (TRACP), while alkaline phosphatase (ALP) remained unchanged. Subgroup analyses indicated higher efficacy in female and naturally aged models, with ginsenosides as active components at doses ≤ 40 mg/(kg·d), and greater effects via intraperitoneal administration. Sensitivity analysis confirmed robustness.

**Conclusion:**

*Panax ginseng* extract demonstrates consistent multi-dimensional anti-osteoporotic effects in animal models, supporting its potential as a multi-target therapeutic candidate. Further studies are warranted to optimize dosage, formulation, and clinical translation.

**Systematic review registration:**

PROSPERO, identifier: CRD420251232252

## Introduction

Osteoporosis (OP) is a systemic skeletal disorder characterized by reduced bone mass and deterioration of bone microarchitecture, which compromise bone strength and increase the risk of fragility fractures (1, 2). This disease represents a major public health concern worldwide (3), affecting more than 200 million people globally (4). Its prevalence varies across countries and continents, ranging from 4.1% in the Netherlands to 52.0% in Turkey, with Africa exhibiting the highest rate at 26.9% (5). Among individuals aged 50 years and older, the annual incidence of osteoporotic fragility fractures typically ranges from 500 to 1,000 cases per 100,000 population (6). Fragility fractures mainly occur at the hip, vertebrae, and distal radius. The incidence of distal radius fractures continues to rise across all age groups and usually occurs earlier than vertebral or hip fractures, providing an opportunity for early intervention (7, 8). However, hip fractures have the most severe consequences, being associated with high morbidity, mortality, and disability rates (9). For example, the 1-year mortality rate among hip fracture patients ranges from 18% to 33%. Moreover, osteoporosis imposes a substantial socioeconomic burden, including both direct medical costs and indirect productivity losses (9, 10).

The pathophysiology of osteoporosis involves a dynamic imbalance in bone remodeling, characterized by enhanced osteoclastic bone resorption and impaired osteoblastic bone formation (11). Key molecular pathways, such as the receptor activator of nuclear factor-κB ligand (RANKL)/RANK/osteoprotegerin (OPG) axis and the Wnt/β-catenin signaling pathway, play central roles in the regulation of bone metabolism (4, 12). Estrogen deficiency, oxidative stress, and a chronic inflammatory microenvironment synergistically contribute to the onset and progression of osteoporosis. Although first-line therapeutic agents such as bisphosphonates and denosumab are widely used, their efficacy remains limited and their long-term safety remains controversial. Adverse events, including atypical femoral fractures and osteonecrosis of the jaw, together with poor patient adherence, limit the effectiveness of current treatment regimens. Therefore, there is an urgent need to develop novel alternative therapies with multi-target actions and low toxicity (13, 14).

As a traditional herbal medicine, *Panax ginseng* has a history of use spanning thousands of years in Asian cultures and has been widely recognized for its diverse pharmacological properties. Currently, it is also one of the most commonly used nutritional supplements worldwide (15). Modern studies have demonstrated that *Panax ginseng* and its major active constituents, ginsenosides, possess significant anti-inflammatory, antioxidant, and immunomodulatory properties (16, 17). These characteristics confer potential benefits in regulating bone metabolism. For instance, ginsenosides ma y suppress osteoclast activity and promote osteoblast function by modulating the RANKL/RANK/osteoprotegerin (OPG) axis, activating the Wnt/β-catenin signaling pathway, and inhibiting oxidative stress and inflammatory microenvironments (18). Furthermore, ginsenoside Rc has been shown to alleviate osteoporosis via activation of the TGF-β/Smad signaling pathway, whereas ginsenoside Rb1 exerts anti-osteoporotic effects through modulation of the AHR/PRELP/NF-κB signaling axis (19, 20).

However, findings from current animal model studies remain inconsistent. Some studies have reported that *Panax ginseng* significantly increases bone mineral density (BMD) and improves bone microarchitecture, whereas others have found no significant effects or even reported dose-dependent variations (21, 22). Meta-analysis, as an important tool for integrating and analyzing data from multiple studies, can minimize research bias and enhance the reliability and translational potential of scientific findings (23). Therefore, this study aimed to conduct a rigorous systematic review and meta-analysis to quantitatively evaluate the true efficacy of *Panax ginseng* extracts in animal models of osteoporosis, assess methodological quality and risk of bias (RoB), and identify effective intervention parameters, thereby providing high-quality evidence to address the current bottlenecks in preclinical-to-clinical translation.

## Methods

This meta-analysis was conducted in strict accordance with the Preferred Reporting Items for Systematic Reviews and Meta-Analyses (PRISMA) guidelines (24) and has been prospectively registered in PROSPERO (CRD420251232252).

### Search strategy

A comprehensive search was conducted across eight Chinese and English databases, including Web of Science, PubMed, Embase, the Foreign Medical Literature Retrieval Service (FMRS), Scopus, China National Knowledge Infrastructure (CNKI), Wanfang Data, and VIP Database. The literature search was independently performed by two reviewers. Both keywords and Medical Subject Headings (MeSH) terms were applied for database searches. The databases were searched from their inception to November 1, 2025, using the following combinations of search terms (Supplementary Appendix 1).

### Inclusion and exclusion criteria

This study adopted a randomized controlled trial (RCT) design to systematically screen and compare the effects of *Panax ginseng* extract vs. saline or placebo (vehicle control) in animal models of osteoporosis. The inclusion criteria were as follows: (a) studies involving successfully established osteoporotic animal models; (b) *in vivo* experimental studies; (c) clearly defined outcome measures with extractable data; and (d) randomized controlled trial design. The exclusion criteria included: (a) studies involving animal models with concomitant bone metabolic disorders; (b) *in vitro* studies or those including combined medication protocols or compound formulations; (c) duplicate publications; and (d) non-original studies such as conference abstracts, reviews, commentaries, or letters to the editor.

### Data extraction

After duplicate records were removed, two investigators independently screened the titles and abstracts of all retrieved records according to the predefined eligibility criteria. Studies considered potentially eligible by either reviewer were then retrieved in full text and assessed independently for final inclusion. No formal blinding of reviewer to author name, journal, or institutional affiliation was applied during study selection. Any discrepancies arising during title/abstract screening, full-text assessment, or final study selection were resolved through discussion, and when consensus could not be reached, a third reviewer adjudicated the decision. Data extraction was independently performed by two reviewers using a standardized data extraction form developed in advance. The extracted information included the first author's name, year of publication, method of osteoporosis model establishment, body weight and age of animals, sample size, intervention details and administration route, experimental duration (with clearly specified time units), and the arithmetic mean and standard deviation (SD) of the primary outcome measures. After independent extraction, the two datasets were cross-checked, and disagreements were resolved by discussion or consultation with a third reviewer when necessary. For numerical data presented only in graphical form, values were visually extracted and reconstructed using GetData Graph Digitizer software (Version 2.26).

### Quality assessment

The SYRCLE's risk of bias (RoB) tool was used to independently assess the methodological quality of the included studies (25). The evaluation covered six domains—selection bias, performance bias, detection bias, attrition bias, reporting bias, and other sources of bias—across 10 specific items. Studies meeting all criteria within each domain were judged as having a low risk of bias; those not meeting the criteria were classified as high risk of bias; and when information was insufficient to permit judgment, the risk of bias was rated as unclear. Any discrepancies between reviewers during the assessment process were resolved through discussion to ensure the accuracy and consistency of the final evaluation.

### Outcome measures

The primary outcome of this study was bone mineral density (BMD). Secondary outcomes included parameters of bone microarchitecture [trabecular number, trabecular thickness, trabecular separation, and bone volume fraction (BV/TV)], biomechanical properties [maximum stress, maximum load, stiffness, and structural model index (SMI)], and biochemical markers of bone metabolism, such as procollagen type I N-terminal propeptide (PINP), estradiol (E2), serum alkaline phosphatase, serum osteocalcin, tartrate-resistant acid phosphatase (TRACP), serum calcium, and serum phosphorus.

### Statistical analysis

Data analysis and visualization were performed using Stata software (Stata SE, Version 18) and Review Manager (RevMan, Version 5.4.0). Heterogeneity among studies was assessed using the *I*^2^ statistic. Model selection considered not only the magnitude of statistical heterogeneity but also the biological and methodological comparability of the included animal studies. In general, random-effects models were preferred when heterogeneity was evident, whereas fixed-effect models were used when heterogeneity was low and study characteristics were sufficiently comparable. Subgroup and sensitivity analyses were conducted to further explore the potential sources of heterogeneity. The main factors examined included model type, animal species, sex, active components, dosage, route of administration, and intervention duration. Sensitivity analysis was performed using the leave-one-out approach to test the robustness of the pooled results. For continuous outcomes, standardized mean differences (SMDs) were used when studies reported the same outcome using different measurement scales, units, anatomical sites, or assay methods, whereas mean differences (MDs) were used when outcomes were reported on the same scale and in the same unit across studies. Corresponding 95% confidence intervals (CIs) were calculated, and a *p*-value <0.05 was considered statistically significant. For the primary outcome with a sufficient number of comparisons, possible small-study effects were additionally explored using funnel plot inspection, together with Egger's regression test and Begg's rank correlation test.

## Results

### Search results

**Figure 1:**
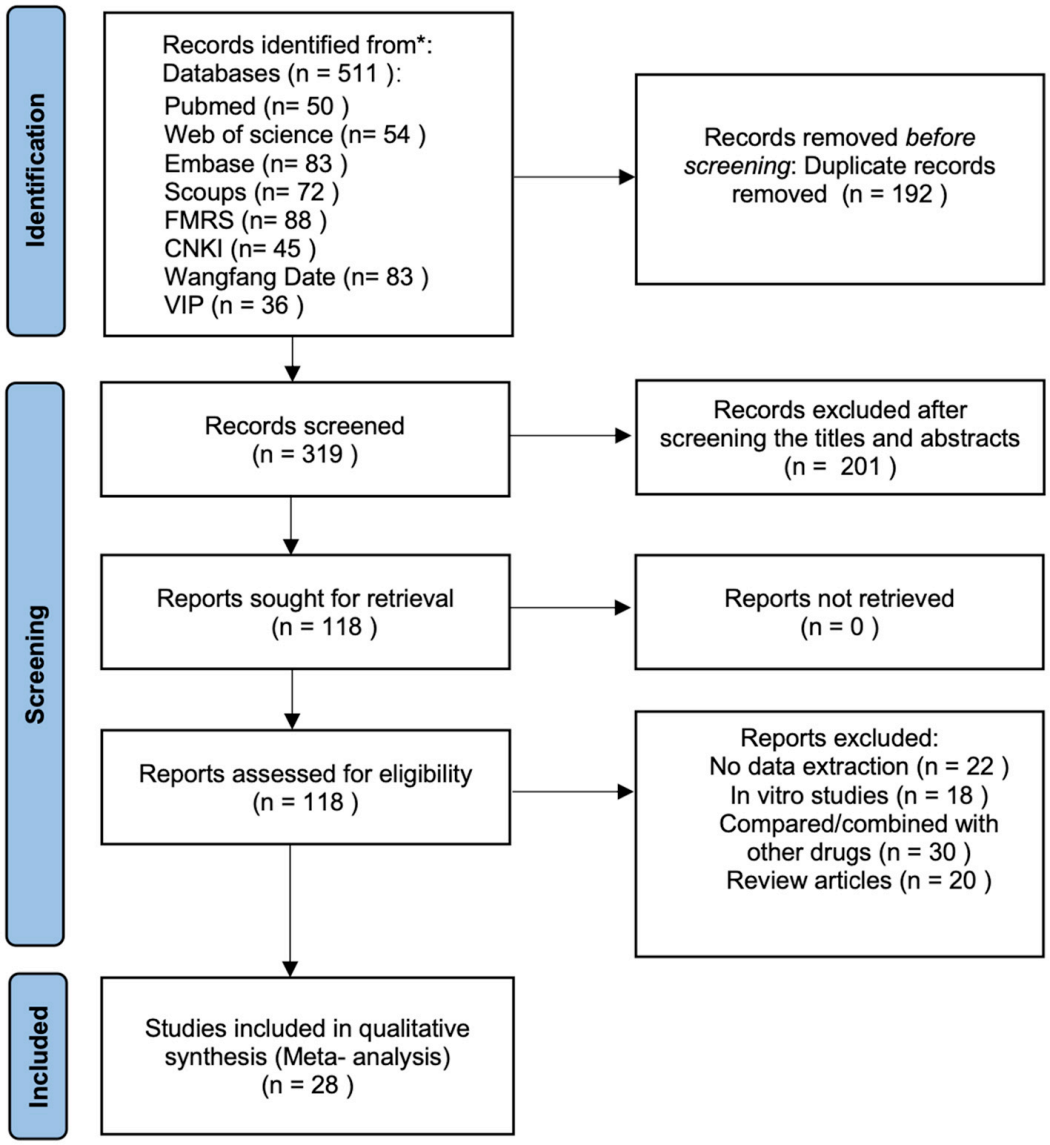
PRISMA flow diagram of study identification, screening, eligibility assessment, and final inclusion. The diagram summarizes the numbers of records retrieved from each database, duplicates removed, records excluded during title/abstract screening, full-text reports assessed for eligibility, reasons for exclusion, and studies ultimately included in the meta-analysis.

The study selection process is illustrated in Figure 1. A total of 511 records were identified through searches of eight Chinese and English databases. After removing 192 duplicates, 319 studies remained. Following title and abstract screening, 201 irrelevant records were excluded. The full texts of the remaining 118 studies were retrieved and reviewed in detail, of which 90 were excluded for the following reasons: (a) 22 studies did not provide extractable data for the primary outcome; (b) 30 studies compared *Panax ginseng* with or combined it with other drugs; (c) 18 were *in vitro* experiments; (d) 20 were review articles. Ultimately, 28 studies were included in the meta-analysis, comprising 8 Chinese-language publications (26–33) and 20 English-language publications (19–22, 34–49).

### Characteristics of included studies

The main characteristics of the included studies are summarized in Table 1. The 28 studies were published between 2004 and 2025. Among these, 15 used ovariectomy (OVX) to induce osteoporosis, 5 employed glucocorticoid induction, and 4 utilized natural aging models. The remaining studies established osteoporosis through retinoic-acid gavage, radiation exposure, aluminum induction, or orchidectomy models. In terms of animal species, 22 studies used rats and 6 used mice. Regarding the active components of *Panax ginseng* extract, all studies reported the source of the bioactive substances. Nineteen studies explicitly identified ginsenosides as the active intervention, eight used aqueous decoction extraction, and one study employed Panax ginseng polysaccharides as the therapeutic agent. The administration routes in the intervention and control groups included oral gavage (21 studies) and injection (7 studies). The dosing range varied from 6 mg/(kg·d) to 1.7 g/(kg·d), with experimental durations spanning 4–84 weeks.

**Table 1:** Characteristics of the included studies.

Reference	Induction of osteoporosis	Species	Gender	Effective substance	Sample size		Intervention		Route of administration	Duration of study	BMD measurement		
					IG	CG	IG	CG			Instrument	Skeletal site	Reported BMD unit
Zhang et al. (31)	RA	SD	Male	*Panax ginseng* extract	10	10	6.62 mg/(kg·d)	DW	Intragastric	4 weeks	DXA	Femur	g/cm^2^
Lv et al. (28)	Glu	Wistar	Female	Ginsenosides	9	9	100 mg/(kg·d)	DW	Intragastric	9 weeks	DXA	Femur	g/cm^2^
Zhou et al. (32)	OVX	Wistar	Female	Ginsenoside Rg3	15	15	80 mg/(kg·d)	PS	Intragastric	12 weeks	DXA	Femur	g/cm^3^
Ma et al. (29)	Natural aging model	SD	Female	Ginsenoside Rg3	10	10	20 mg/(kg·d)	PS	Intragastric	12 weeks	Micro-CT	Femur	g/cm^2^
Guo et al. (26)	Natural aging model	SD	Male	Ginsenosides	10	10	40 mg/(kg·d)	PS	Intragastric	84 weeks	DXA	Tibia	g/cm^2^
Yu et al. (30)	OVX	SD	Female	Ginsenosides	10	10	60 mg/(kg·d)	PS	Intragastric	12 weeks	DXA	Femur	g/cm^2^
Lee et al. (40)	OVX	Wistar	Female	*Panax ginseng* extract	16	16	500 mg/(kg·d)	PS	Intragastric	8 weeks	DXA	Femur	g/cm^2^
Li et al. (27)	OVX	SD	Female	Ginseng polysaccharides	10	10	200 mg/(kg·d)	DW	Intragastric	12 weeks	DXA	Femur	g/cm^2^
Gong et al. (36)	OVX	Wistar	Female	Ginsenosides	8	8	40 mg/(kg·d)	PS	Intragastric	24 weeks	DXA	Tibia	g/cm^2^
Lee et al. (41)	Radiation-induced	C3H/HeN	Female	*Panax ginseng* extract	6	6	250 mg/(kg·d)	PS	Intragastric	12 weeks	Micro-CT	Tibia	g/cm^3^
Peng et al. (43)	Glu	Wistar	Female	Ginsenoside Rg3	15	15	10 mg/(kg·d)	PS	Intragastric	12 weeks	DXA	Femur	g/cm^2^
Chen et al. (22)	OVX	C57BL/J	Female	Ginsenoside Rg1	14	14	20 mg/(kg·d)	PS	Subcutaneous	12 weeks	Micro-CT	Femur	g/cm^3^
Yang et al. (45)	OVX	C3H/HeN	Female	*Panax ginseng* extract	6	6	250 mg/(kg·d)	PS	Intragastric	6 weeks	Micro-CT	Tibia	g/cm^3^
Yang et al. (46)	OVX	C57BL/J	Female	Ginsenosides	12	12	50 mg/(kg·d)	PS	Intragastric	12 weeks	Micro-CT	Femur	g/cm^3^
Chen et al. (35)	Glu	Wistar	Female	Ginsenoside Rg1	10	10	110 mg/(kg·d)	PS	Intragastric	12 weeks	DXA	Femur	g/cm^2^
Song et al. (44)	Aluminum-induced	SD	Male	Ginsenoside Rg3	10	10	20 mg/(kg·d)	PS	Intragastric	4 weeks	DXA	Femur	g/cm^2^
Huang et al. (37)	OVX	BALB/c	Female	Ginsenoside Rb2	10	10	18.5 μmol/(kg·d)	PS	Intraperitoneal	12 weeks	Micro-CT	Femur	g/cm^3^
Zhang et al. (47)	Glu	SD	Female	Ginsenoside Rg3	8	8	20 mg/(kg·d)	PS	Intragastric	5 weeks	DXA	Femur	g/cm^2^
Kim et al. (39)	Natural aging model	Wistar	Male	*Panax ginseng* extract	10	10	300 mg/(kg·d)	PS	Intragastric	8 weeks	DXA	Femur	g/cm^2^
Zhang et al. (48)	OVX	SD	Female	Ginsenoside Rg3	12	12	20 mg/(kg·d)	PS	Intraperitoneal	5 weeks	Micro-CT	Femur	g/cm^3^
Zhang et al. (20)	Glu	SD	Female	Ginsenoside Rb1	6	6	6 mg/(kg·d)	PS	Intraperitoneal	12 weeks	DXA	Femur	g/cm^2^
Ma et al. (42)	Testicular type	C57BL/J	Male	Ginsenoside Rb2	6	6	10 mg/(kg·d)	PS	Intraperitoneal	8 weeks	DXA	Femur	g/cm^2^
Wang et al. (19)	OVX	SD	Female	Ginsenosides	10	10	20 mg/(kg·d)	PS	Intraperitoneal	4 weeks	DXA	Femur	g/cm^2^
Bei et al. (21)	OVX	SD	Female	Ginsenoside Rb1	8	8	6 mg/(kg·d)	PS	Intraperitoneal	12 weeks	DXA	Femur	g/cm^2^
Avsar et al. (34)	OVX	Wistar	Female	*Panax ginseng* extract	8	8	200 mg/(kg·d)	PS	Intragastric	4 weeks	DXA	Femur	g/cm^2^
Zhu et al. (33)	Natural aging model	SD	Female	Ginsenoside Rh2	10	10	300 mg/(kg·d)	PS	Intragastric	12 weeks	Micro-CT	Femur	g/cm^3^
Zhang et al. (49)	OVX	SD	Female	*Panax ginseng* extract	8	8	600 mg/(kg·d)	PS	Intragastric	12 weeks	Micro-CT	Femur	g/cm^3^
Jung et al. (38)	OVX	SD	Female	*Panax ginseng* extract	7	7	1.7 g/(kg·d)	PS	Intragastric	8 weeks	Micro-CT	Femur	g/cm^3^

OVX, ovariectomy; Glu, glucocorticoid; RA, retinoic acid; IG, intervention group; CG, control group; PS, physiological saline; DW, distilled water; DXA, dual-energy X-ray absorptiometry.

### Quality assessment of included studies

**Figure 2:**
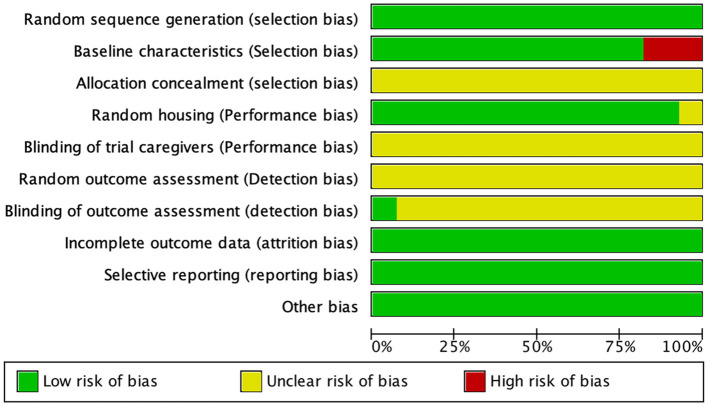
Summary of risk-of-bias assessment for the included studies using the SYRCLE tool. The bar chart shows the proportion of studies judged as low, unclear, or high risk of bias across the assessed domains, including sequence generation, baseline characteristics, allocation concealment, random housing, blinding of caregivers, random outcome assessment, blinding of outcome assessment, incomplete outcome data, selective reporting, and other bias.

The risk of bias for the included studies was assessed using the SYRCLE tool, and the results are presented in Figure 2. All studies reported random sequence generation and showed no evidence of incomplete outcome data, selective reporting, or other sources of bias. However, important methodological safeguards were insufficiently reported in several domains. Allocation concealment and blinding of caregivers were not described in the included studies, and only two studies reported blinding during outcome assessment. In addition, two studies did not specify random housing procedures, and four studies did not fully report baseline characteristics. Overall, these findings indicate important limitations in methodological reporting and suggest possible risk of performance and detection bias in the included evidence base.

### BMD and subgroup analyses

**Figure 3:**
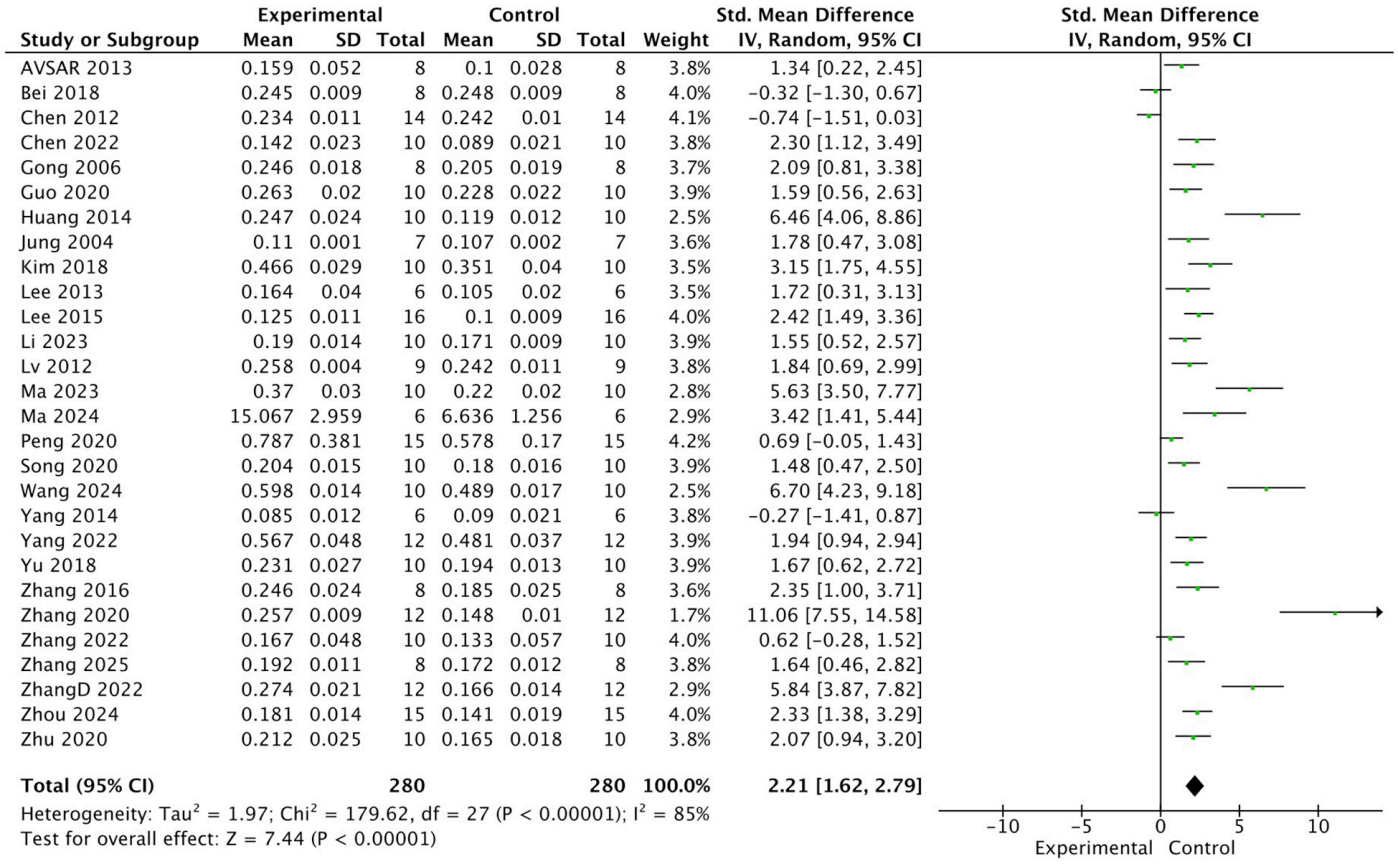
Forest plot of the effect of *Panax ginseng* extract on bone mineral density (BMD) in osteoporosis animal models. Effect sizes are presented as standardized mean differences (SMDs) with 95% confidence intervals (CIs) under a random-effects model.

This study, including data from 28 experiments, confirmed the efficacy of *Panax ginseng* extract in improving bone mineral density (BMD) in osteoporosis animal models, as shown in Figure 3. The BMD in the *Panax ginseng*-treated groups was significantly higher than that in the control groups [standardized mean difference (SMD) = 2.21, 95% *CI* = 1.62 to 2.79, *p* < 0.000001). Subgroup analyses were conducted based on model type, species, sex, active components, dosage, route of administration, measuring instrument, skeletal site and intervention duration to explore potential sources of heterogeneity, with results presented in Table 2. We observed that *I*^2^ values decreased notably for sex, active components, dosage, and administration route, suggesting that these factors may contribute to the observed heterogeneity. Furthermore, *Panax ginseng* showed greater efficacy in natural aging models, in female animals, when ginsenosides were used as the active component, at doses ≤ 40 mg/(kg·d), and via intraperitoneal administration.

**Table 2:** Subgroup analysis of bone mineral density based on multiple factors related to *Panax ginseng* treatment.

Subgroup	Standardized mean difference (95% confidence interval)	*I* ^2^	*p*-value
Model			
OVX	2.17 [1.26, 3.08]	89	0.000
GLU	2.42 [1.06, 3.77]	85	0.000
Natural aging model	2.87 [1.49, 4.25]	76	0.000
Other	1.55 [0.60, 2.49]	57	0.000
Species			
Rat	2.28 [1.69, 2.86]	81	0.000
Mice	1.86 [0.19, 3.54]	91	0.000
Gender			
Male	1.85 [0.91, 2.79]	68	0.000
Female	2.30 [1.61, 2.99]	87	0.000
Effective substance			
*Panax ginseng* extract	1.51 [0.79, 2.24]	69	0.000
Ginsenosides	2.65 [1.82, 3.48]	89	0.000
Dose			
≤ 40 mg/(kg·d)	2.92 [1.76, 4.07]	91	0.000
>40 mg/(kg·d)	1.81 [1.43, 2.20]	39	0.000
Methods of administration			
Intragastric	1.77 [1.39, 2.15]	59	0.000
Intraperitoneal	5.37 [2.00, 8.74]	94	0.000
Duration of intervention			
≤ 9 weeks	2.47 [1.52, 3.42]	84	0.000
>9 weeks	2.04 [1.29, 2.80]	86	0.000
Measurement instrument			
DXA	2.05 [1.48, 2.62]	79	0.000
Micro-CT	2.72 [1.32, 4.13]	92	0.019
Measuring position			
Femur	2.43 [1.76, 3.09]	87	0.000
Tibia	1.27 [0.21, 2.32]	68	0.000

### Bone histomorphometry

**Figure 4:**
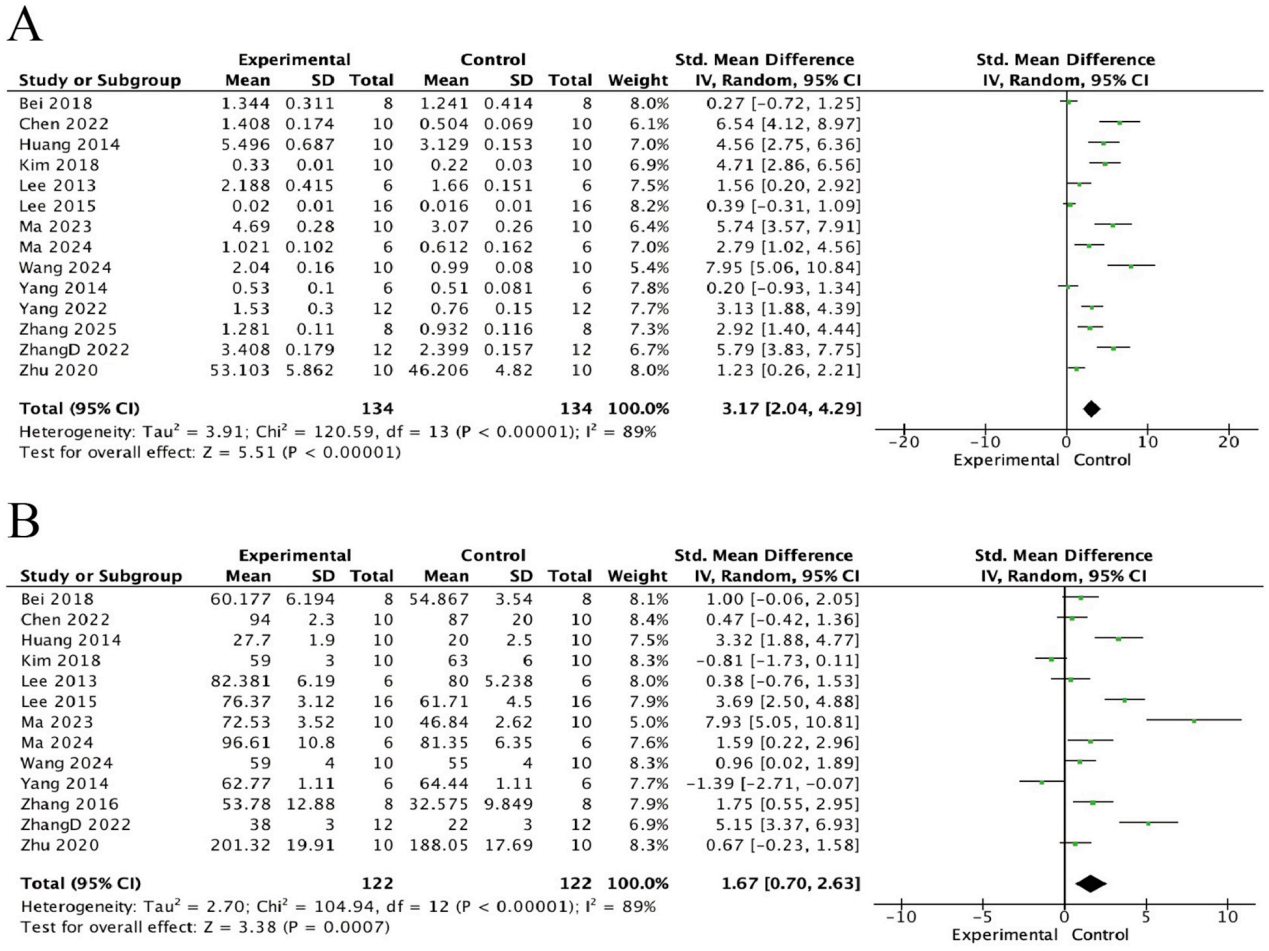
Forest plots of the effects of *Panax ginseng* extract on bone microarchitecture parameters. **(A)** Trabecular number (Tb.N). **(B)** Trabecular thickness (Tb.Th). Effect sizes are presented as standardized mean differences (SMDs) with 95% confidence intervals (CIs) under random-effects models.

**Figure 5:**
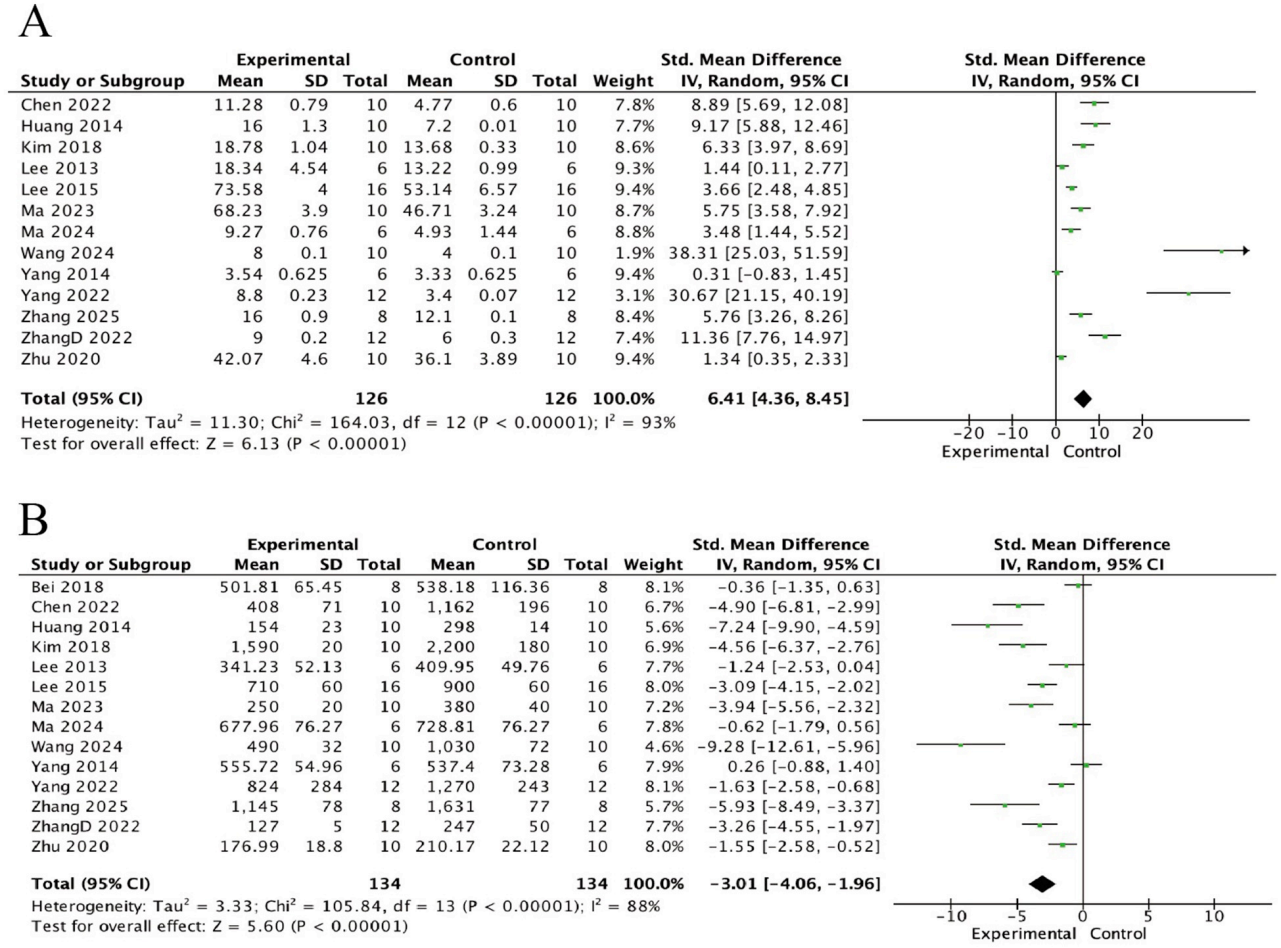
Forest plots of the effects of *Panax ginseng* extract on additional bone microarchitecture parameters. **(A)** Bone volume fraction (BV/TV). **(B)** Trabecular separation (Tb.Sp). Effect sizes are presented as standardized mean differences (SMDs) with 95% confidence intervals (CIs) under random-effects models.

The meta-analysis of the effects of *Panax ginseng* extract on bone microarchitecture in osteoporosis animal models is presented in Figures 4, 5. In Figure 4, 14 studies reported the effects of *Panax ginseng* extract on trabecular number, showing a significant increase (*SMD* = 3.17, 95% *CI* = 2.04 to 4.29, *p* < 0.000001). Thirteen studies demonstrated a significant improvement in trabecular thickness following *Panax ginseng* treatment (*SMD* = 1.67, 95% *CI* = 0.70 to 2.63, *p* < 0.000001). Figure 5 illustrates the effects of *Panax ginseng* extract on bone volume fraction (BV/TV) and trabecular separation. Thirteen studies reported a significant increase in BV/TV (*SMD* = 6.41, 95% *CI* = 4.36 to 8.45, *p* < 0.000001), while 14 studies showed a significant reduction in trabecular separation (*SMD* = −3.01, 95% *CI* = −4.06 to −1.96, *p* < 0.000001).

### Bone biomechanical parameters

**Figure 6:**
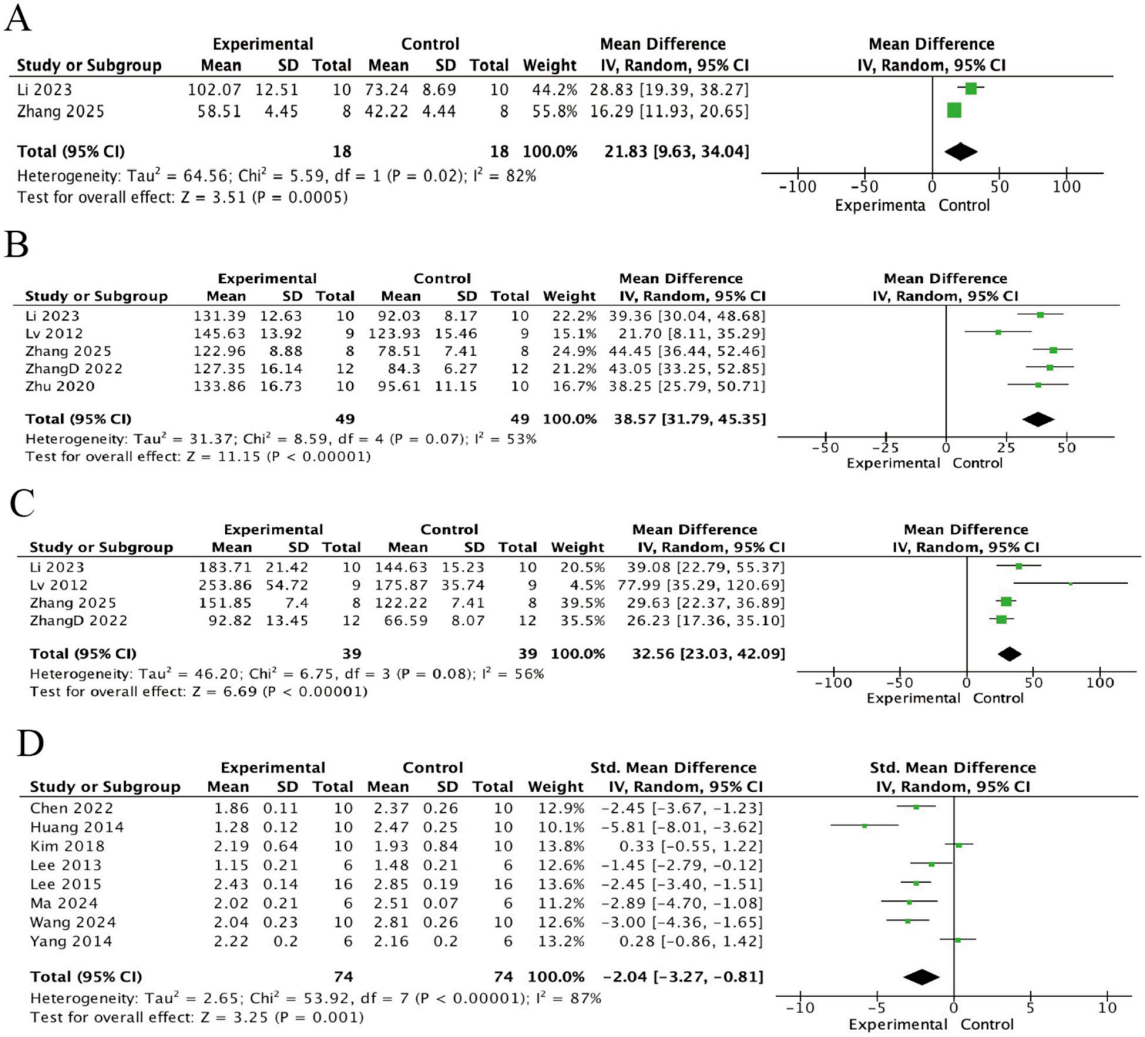
Forest plots of the effects of *Panax ginseng* extract on bone biomechanical properties. **(A)** Ultimate stress. **(B)** Maximum load. **(C)** Stiffness. **(D)** Structural model index (SMI). Mean differences (MDs) were used for ultimate stress, maximum load, and stiffness because these outcomes were pooled on directly comparable scales; standardized mean differences (SMDs) were used for SMI. All results are shown with 95% confidence intervals (CIs).

The meta-analysis of the effects of *Panax ginseng* extract on bone biomechanical parameters in osteoporosis animal models is presented in Figure 6. Two studies reported results for ultimate stress (*MD* = 21.83, 95% *CI* = 9.63 to 34.04, *p* = 0.0005), and five studies reported results for maximum load (*MD* = 38.57, 95% *CI* = 31.79 to 45.35, *p* < 0.000001). Four studies reported stiffness outcomes (*MD* = 32.56, 95% *CI* = 23.03 to 42.09, *p* < 0.000001), and eight studies reported the structural model index (SMI; SMD = −2.04, 95% *CI* = −3.27 to −0.81, *p* = 0.001).

### Bone metabolism markers

**Figure 7:**
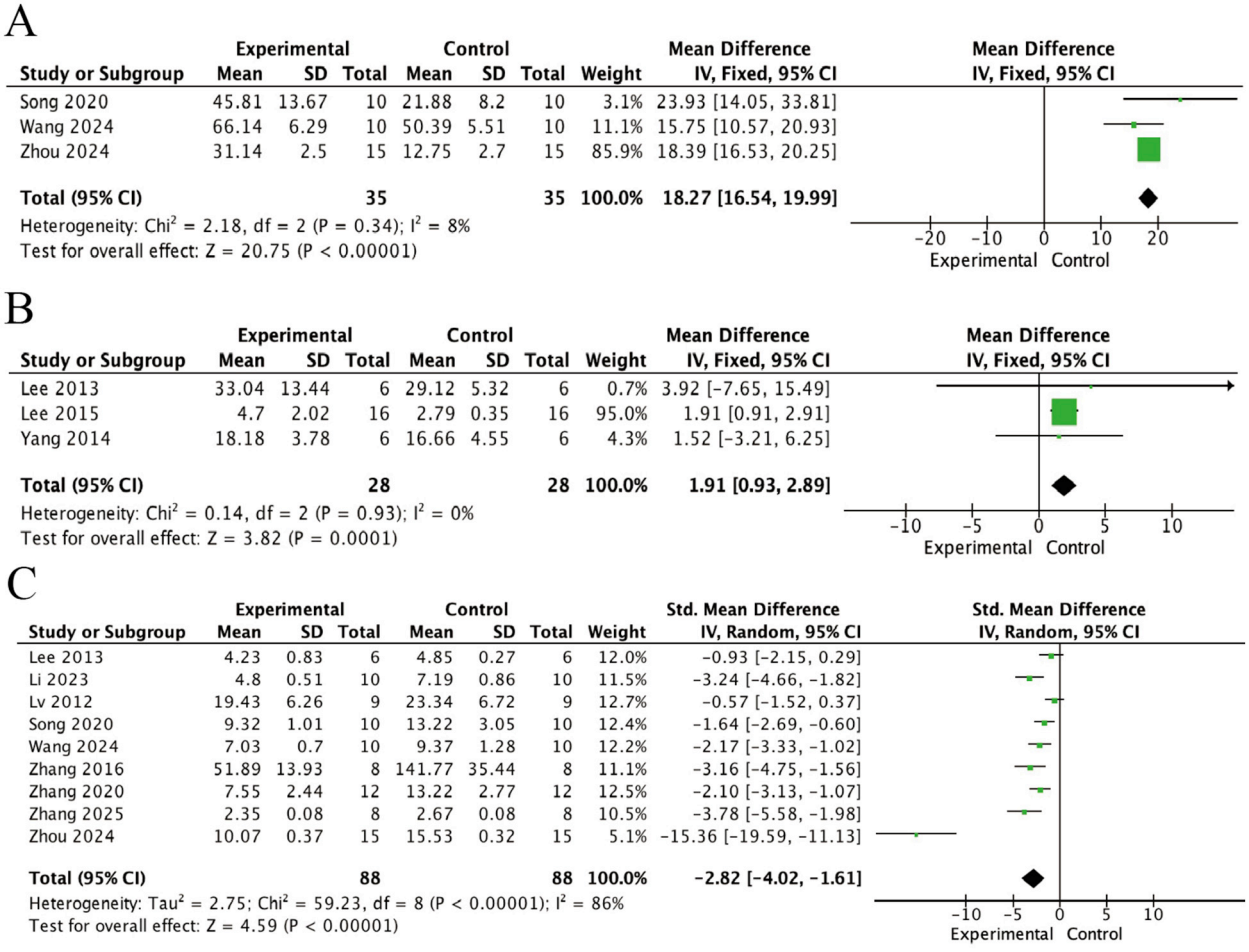
Forest plots of the effects of *Panax ginseng* extract on biochemical markers of bone metabolism. **(A)** Procollagen type I N-terminal propeptide (PINP). **(B)** Estradiol (E2). **(C)** Tartrate-resistant acid phosphatase (TRACP). Mean differences (MDs) were used for PINP and E2, whereas standardized mean differences (SMDs) were used for TRACP. Results are shown with 95% confidence intervals (CIs).

**Figure 8:**
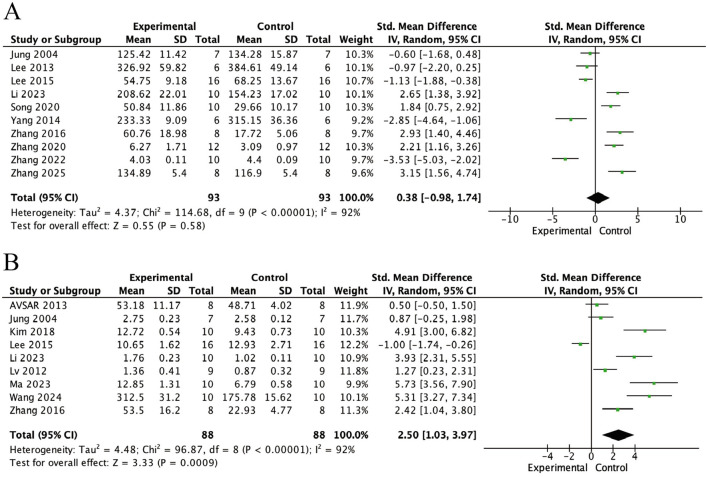
Forest plots of the effects of *Panax ginseng* extract on serum alkaline phosphatase (ALP) and osteocalcin (OC). **(A)** ALP. **(B)** OC. Effect sizes are presented as standardized mean differences (SMDs) with 95% confidence intervals (CIs) under random-effects models.

**Figure 9:**
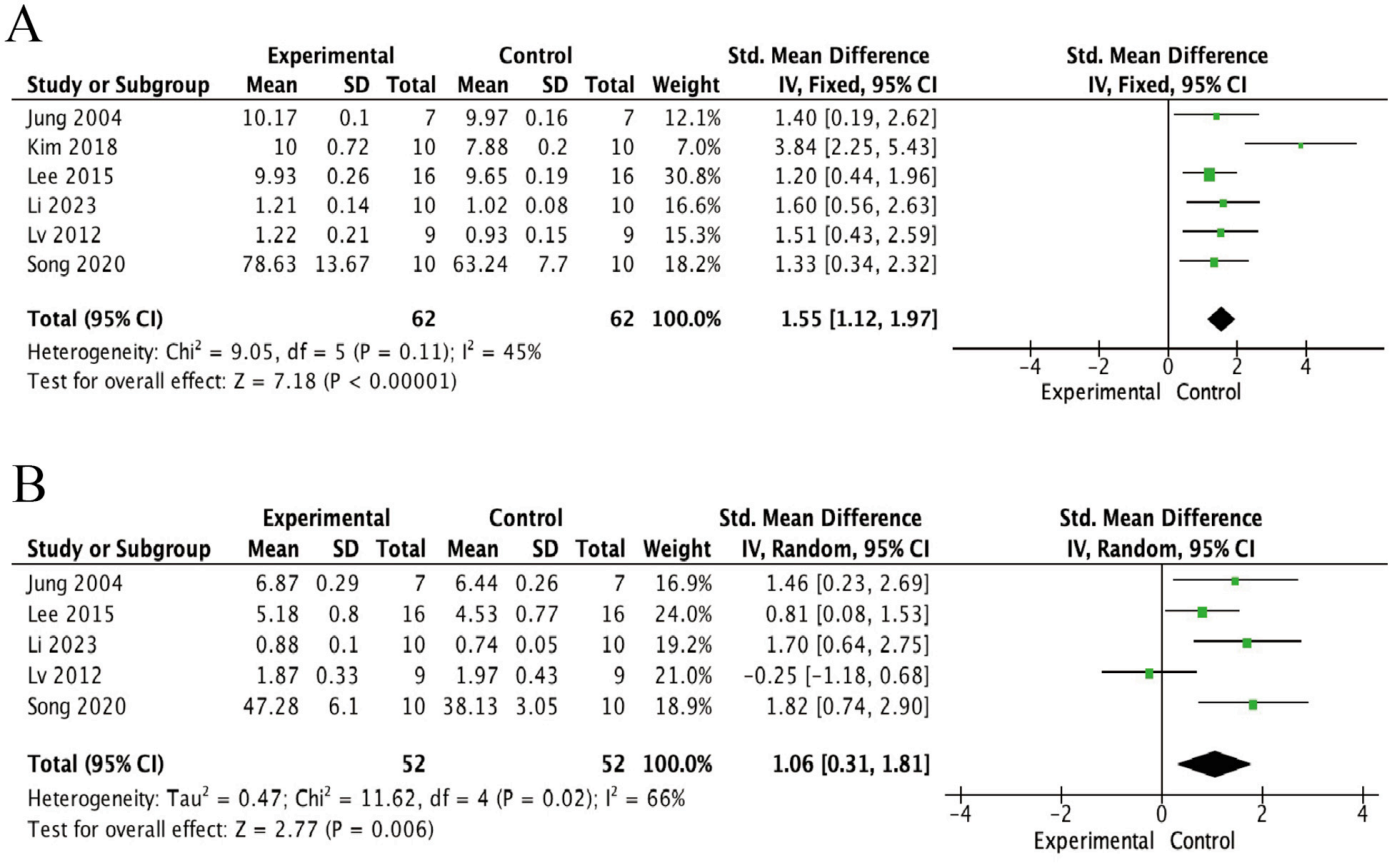
Forest plots of the effects of *Panax ginseng* extract on mineral metabolism indicators. **(A)** Serum calcium. **(B)** Serum phosphate. Effect sizes are presented as standardized mean differences (SMDs) for serum calcium and serum phosphate, with 95% confidence intervals (CIs).

Figures 7–9 collectively present the meta-analysis results of *Panax ginseng* extract on bone metabolism and biochemical markers in osteoporosis animal models. In Figure 7, three studies reported procollagen I N-terminal propeptide (PINP) levels (*MD* = 18.27, 95% *CI* = 16.54 to 19.99, *p* < 0.000001), three studies reported serum estradiol (E2) levels (*MD* = 1.91, 95% *CI* = 0.93 to 2.89, *p* = 0.0001), and nine studies reported tartrate-resistant acid phosphatase (TRACP) outcomes (*SMD* = −2.82, 95% *CI* = −4.02 to −1.61, *p* < 0.0001). Figure 8 presents the meta-analysis of alkaline phosphatase (ALP) and osteocalcin (OC). Ten studies reported ALP outcomes comparing *Panax ginseng* extract with control interventions (*SMD* = 0.38, 95% *CI* = −0.98 to 1.74, *p* = 0.58), showing no statistical significance. Nine studies reported OC outcomes (*SMD* = 2.50, 95% *CI* = 1.03 to 3.97, *p* = 0.0009), indicating a significant effect. Figure 9 shows the meta-analysis of serum calcium (Ca) and phosphate (P). Six studies reported serum Ca levels (*SMD* = 1.55, 95% *CI* = 1.12 to 1.97, *p* < 0.00001), and five studies reported serum P levels (*SMD* = 1.06, 95% *CI* = 0.31 to 1.81, *p* = 0.006), both showing statistically significant differences.

### Sensitivity analysis results and publication bias

**Figure 10:**
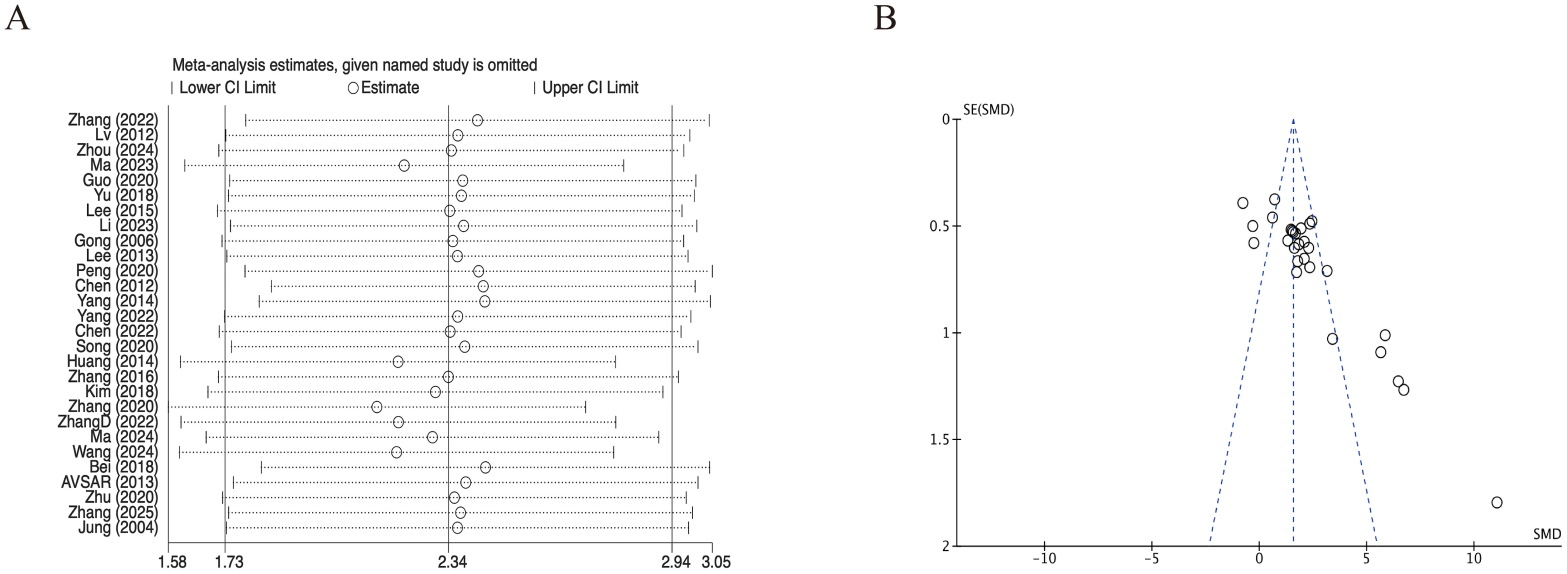
Sensitivity analysis and funnel plot. **(A)** Leave-one-out sensitivity analysis. The horizontal axis represents the pooled effect size after omitting each individual study, expressed as the standardized mean difference (SMD) with its 95% confidence interval. The vertical axis lists the omitted studies. **(B)** Funnel plot for assessing publication bias. The horizontal axis represents the standardized mean difference (SMD), and the vertical axis represents the standard error of the SMD [SE(SMD)]. Because SMD is a standardized effect-size measure, both SMD and SE(SMD) are dimensionless.

Sensitivity analysis was conducted for the primary BMD outcome using the leave-one-out approach, and the results are presented in Figure 10A. Sequential omission of each included study did not materially alter the pooled effect estimate, indicating that the overall result was not disproportionately driven by any single study. The pooled estimates remained within a relatively narrow range, and the overall direction of effect was unchanged, supporting the stability of the primary meta-analytic finding. Because the primary BMD dataset included a sufficient number of comparisons, possible small-study effects were further explored using funnel plot inspection (Figure 10B). Visual inspection of the funnel plot suggested apparent asymmetry, with several less precise studies tending to show relatively larger effect sizes. In addition, both Egger's regression test and Begg's rank correlation test were significant (both *p* < 0.05), suggesting possible small-study effects. Therefore, although the primary BMD result appeared stable in sensitivity analysis, it should still be interpreted with appropriate caution.

## Discussion

This study is the first to quantitatively assess the overall efficacy of *Panax ginseng* extract in osteoporosis animal models through a systematic review and meta-analysis, encompassing multiple dimensions including bone mineral density, microarchitecture, biomechanical properties, and bone metabolism. The core findings indicate that *Panax ginseng* extract significantly increases BMD (*SMD* = 2.21), improves trabecular parameters, and enhances bone biomechanical performance, with effect sizes comparable to or even exceeding those of certain conventional anti-osteoporotic agents in animal models (50, 51). These conclusions were confirmed to be highly robust through sensitivity analysis, providing evidence-based support for overcoming the preclinical translational barriers of *Panax ginseng* formulations. The study further revealed that model type, route of administration, and composition of active components are key modifiers of efficacy, highlighting critical considerations for optimizing experimental design and guiding clinical translation.

The biological basis of efficacy is rooted in the synergistic regulation of bone remodeling networks by multiple components of *Panax ginseng*. Existing mechanistic studies have demonstrated that ginsenosides Rb1 and Rc inhibit osteoclast differentiation and promote osteogenesis via the AHR/PRELP/NF-κB and TGF-β/Smad pathways, respectively (19, 20), which corroborates the present meta-analysis findings of significant reductions in TRACP and concurrent increases in OC and PINP. Notably, serum alkaline phosphatase (ALP) did not show statistically significant improvement, suggesting that *Panax ginseng* extract may promote matrix mineralization via non-classical pathways rather than through a systemic upregulation of ALP (52). This “decoupling” phenomenon indicates that the osteogenic effects of *Panax ginseng* are tissue-specific, and future studies should distinguish between bone-derived and liver-derived ALP isoenzymes to precisely elucidate the sites of action (53). Furthermore, the antioxidant and anti-inflammatory properties of *Panax ginseng* can mitigate oxidative stress in the osteoporotic microenvironment (16, 18, 54). This multi-target profile may offer potential advantages over single-pathway inhibitors such as bisphosphonates, which mainly inhibit bone resorption without directly reversing deficits in bone formation (51, 55).

The effect modifiers identified through subgroup analysis warrant detailed interpretation. The efficacy observed in naturally aged models exceeded that in OVX models (with a notable decrease in *I*^2^), suggesting that *Panax ginseng* may be more advantageous for age-related composite bone loss, potentially through mitigation of systemic inflammaging (18, 46). The stronger effects in female animal subgroups align with the pathophysiological context dominated by estrogen deficiency, and *Panax ginseng's* weak estrogenic activity along with its capacity to elevate E2 levels may partially account for this observation (56). Dose-response analysis indicated that a threshold dose of ≤ 40 mg/(kg·d) achieved maximal efficacy, with higher doses providing no additional benefit. This is consistent with the biphasic dose-response characteristics of ginsenosides and provides a safety margin for clinical dose optimization (46, 57). Notably, differences in the route of administration were observed: the intraperitoneal group exhibited significantly greater effect sizes than the oral gavage group, reflecting the limitation of low oral bioavailability. Ginsenosides are readily metabolized by gut microbiota and subject to first-pass effects, resulting in less than 10% absorption of the parent compounds, whereas nanovesicle or liposomal encapsulation techniques can enhance oral absorption by 3–5-fold (58, 59). Therefore, pharmaceutical formulation improvements may be a key strategy to achieve oral bioequivalence.

Compared with existing literature, this study is the first to integrate dispersed animal experimental data from an evidence-based perspective. The observed effect size (*SMD* = 2.21) exceeds the mean range reported in previous single-center studies, attributable to the enhanced statistical power and heterogeneity adjustment provided by meta-analysis.

However, direct comparisons with conventional agents should be interpreted cautiously: although bisphosphonates have been clinically proven to reduce fracture risk, their effect sizes in animal models are often limited by species differences and inadequate simulation of dosing regimens (60, 61). In addition, the currently included studies did not systematically assess adverse effects or toxicological endpoints. Therefore, the safety profile of *Panax ginseng* extract remains uncertain, and any presumed safety advantage over conventional anti-osteoporotic agents should be interpreted cautiously until supported by dedicated toxicological and long-term studies. Furthermore, the pooled effects on bone biomechanical parameters (e.g., maximum load *MD* = 38.57) align with trends reported in recent single-component studies; however, the limited number of included studies (*n* = 2–8) underscores the need to expand sample sizes to validate the clinical translational potential of structural strength improvements.

Several limitations of this study should be considered when interpreting the findings. First, methodological limitations in the included animal studies may have introduced bias. The SYRCLE assessment showed that allocation concealment and blinding of caregivers were generally not reported, and only a small minority of studies described blinding of outcome assessment. Such insufficient reporting of key methodological safeguards raises concern about possible performance and detection bias and may have contributed to overestimation of treatment effects. Although sensitivity analyses supported the directional stability of the primary findings, these results should still be interpreted with caution. Future studies should rigorously follow the ARRIVE guidelines to enhance the quality of preclinical evidence (62, 63). Second, residual heterogeneity remains: although subgroup analyses identified partial sources, I^2^ values for most outcomes remained >75%, reflecting unquantified variations in extraction procedures, ginsenoside composition, and animal strains. Future studies are recommended to mandate HPLC fingerprinting and component quantification to enable reproducible pharmacodynamic comparisons. Third, the external validity of the included models remains limited. Most studies used young adult ovariectomized animals and relatively short intervention periods (median approximately 8 weeks), which restricts assessment of long-term efficacy, delayed or chronic toxicity, and the applicability of these findings to human osteoporosis. Therefore, the present results should be interpreted primarily as evidence of short- to medium-term preclinical efficacy rather than definitive support for long-term therapeutic benefit or clinical translation. Fourth, mechanistic evidence remains sparse: only a few studies simultaneously measured signaling pathway molecules, preventing meta-regression from quantifying the contribution of specific mechanisms and limiting the derivation of common biological principles. Fifth, possible small-study effects should also be acknowledged. For the primary BMD outcome, funnel plot inspection suggested apparent asymmetry, and formal statistical testing was consistent with possible small-study effects. Nevertheless, such asymmetry in preclinical animal meta-analyses should be interpreted cautiously, because it may reflect not only dissemination bias but also limited sample size, heterogeneous experimental design, selective reporting, and the statistical behavior of standardized mean differences. Accordingly, while the primary result remained directionally stable in sensitivity analysis, the magnitude of the pooled BMD estimate may still have been influenced by small-study effects and should therefore be interpreted with caution.

Looking ahead, three directions may advance *Panax ginseng* research against osteoporosis from an evidence-based approach toward precision applications. First, integrating multi-omics technologies (transcriptomics, metabolomics) can elucidate the systemic regulatory networks of *Panax ginseng* on the bone–immune–metabolic axis and identify predictive biomarkers of therapeutic efficacy (64, 65). Second, the development of bone-targeted delivery systems (e.g., hydroxyapatite-modified nanoparticles) may enhance local drug concentration while reducing systemic exposure (66, 67). Finally, exploratory clinical trials targeting postmenopausal patients with osteoporosis who are intolerant to conventional therapy should be initiated, employing adaptive designs to optimize dosage and formulation progressively and to assess the risk–benefit profile in real-world settings. In summary, this meta-analysis confirms the scientific value of *Panax ginseng* extracts as multi-target anti-osteoporotic candidates, and their clinical translational potential depends on standardized preparation, mechanistic elucidation, and the establishment of a high-quality clinical evidence chain.

## Data Availability

The original contributions presented in the study are included in the article/Supplementary material, further inquiries can be directed to the corresponding author.
