# Supplementary material for: Nutritional supplementation with Panax ginseng extract and bone health in osteoporotic animal models: a systematic review and meta-analysis
Source: Front Nutr. 2026 Jun 3;13:1748861. doi: 10.3389/fnut.2026.1748861 (PMC13271931; doi:10.3389/fnut.2026.1748861)
Supplement: Supplementary file 1 [file Table_1.DOCX]

**Pubmed**

("Osteoporosis"[Mesh] OR osteoporosis*[tiab] OR "bone loss"[tiab] OR "bone mineral density"[tiab]) AND ("Panax ginseng"[Mesh] OR "Panax ginseng"[tiab] OR ginseng*[tiab] OR ginsenoside*[tiab]) AND (rat[tiab] OR rats[tiab] OR mouse[tiab] OR mice[tiab] OR murine[tiab] OR rodent*[tiab] OR "Animals"[Mesh] NOT humans[MeSH])

**Web of science**

TS=(osteoporos* OR "bone loss" OR "bone mineral densit*") AND TS=(ginseng* OR ginsenoside* OR "Panax ginseng") AND TS=(rat OR rats OR mouse OR mice OR murine OR rodent* OR rodents OR animal* OR "animal model*") NOT TS=(human* OR patient* OR volunteer*)

**Embase**

('osteoporosis'/exp OR osteoporosis:ti,ab OR 'bone loss':ti,ab OR 'bone mineral density':ti,ab) AND

('Panax ginseng'/exp OR 'Panax ginseng':ti,ab OR ginseng*:ti,ab OR ginsenoside*:ti,ab) AND (rat:ti,ab OR rats:ti,ab OR mouse:ti,ab OR mice:ti,ab OR murine:ti,ab OR rodent*:ti,ab OR 'animal'/exp OR 'nonhuman'/exp) NOT 'human'/exp

**FRMS**

("Osteoporosis" OR "osteoporosis" OR "bone loss" OR "bone mineral density") AND ("Panax ginseng" OR "Panax ginseng" OR "ginseng" OR "ginsenoside") AND ("rat" OR "rats" OR "mouse" OR "mice" OR "murine" OR "rodent" OR "Animals" NOT "humans")

**Scoups**

(TITLE-ABS("osteoporosis" OR "bone loss" OR "bone mineral density") AND TITLE-ABS("Panax ginseng" OR "ginseng" OR "ginsenoside") AND (TITLE-ABS("rat" OR "rats" OR "mouse" OR "mice" OR "murine" OR "rodent") AND NOT TITLE-ABS("human" OR "humans")))

**CNKI (China National Knowledge Infrastructure)**

| **Search #** | **Search Concepts** | **Search Strategy** |
| --- | --- | --- |
| #1 | Ginseng-related terms | 主题 = (人参 OR 红参 OR 人参提取物 OR 人参皂苷 OR 人参总皂苷 OR "Panax ginseng") |
| #2 | Osteoporosis-related terms | 主题 = (骨质疏松 OR 骨密度 OR 骨丢失 OR 骨代谢) |
| #3 | Animal models | 主题 = (大鼠 OR 小鼠 OR 动物 OR 鼠类 OR 骨质疏松模型) |
| #4 | Combination | #1 AND #2 AND #3 |

**Wanfang Data**

| Search # | Search Concepts | Original Search Strategy (Chinese) |
| --- | --- | --- |
| #1 | Ginseng-related terms | 主题: (人参 OR 红参 OR 人参提取物 OR 人参皂苷 OR 人参总皂苷 OR "Panax ginseng") |
| #2 | Osteoporosis-related terms | 主题: (骨质疏松 OR 骨密度 OR 骨丢失 OR 骨代谢) |
| #3 | Animal models | 主题: (大鼠 OR 小鼠 OR 动物 OR 鼠类 OR 骨质疏松模型) |
| #4 | Combination | #1 AND #2 AND #3 |

**VIP Database (Chinese Science and Technology Periodical Database)**

| **Search #** | **Search Concepts** | **Search Strategy** |
| --- | --- | --- |
| #1 | Ginseng-related terms | 题名或关键词:(人参 OR 红参 OR 人参提取物 OR 人参皂苷 OR 人参总皂苷 OR "Panax ginseng" OR ginsenoside*) |
| #2 | Osteoporosis-related terms | 题名或关键词:(骨质疏松 OR 骨密度 OR 骨丢失 OR 骨代谢 OR osteoporosis OR "bone mineral density" OR "bone loss" OR "bone metabolism") |
| #3 | Animal models | 题名或关键词:(大鼠 OR 小鼠 OR 动物 OR 鼠类 OR 骨质疏松模型 OR rat OR rats OR mouse OR mice OR animal* OR rodent*) |
| #4 | Combination | #1 AND #2 AND #3 |
